# Comparative analysis of implant survival, peri-implant health, and patient satisfaction among three treatment modalities in atrophic posterior mandibles: a randomized clinical study

**DOI:** 10.1186/s12903-025-06286-7

**Published:** 2025-06-07

**Authors:** Eman Mohamed Raffat, Mohamed Shady, Ayman Abdel Rahim Elkashty, Moustafa El Syad

**Affiliations:** 1 Prosthodontic Department, Faculty of Dentistry, Mansoura University, Mansoura, Egypt; 2 Prosthodontic Department, Faculty of Dentistry, Benha National University, Obour, Egypt; 3 Periodontology, Oral Diagnosis and Radiology Department, Faculty of Dentistry, Mansoura University, Mansoura, Egypt

**Keywords:** Atrophic mandible, Fixed implant prosthesis, Implant survival, Peri-implantitis, Ridge augmentation

## Abstract

**Background:**

This study assessed the clinical outcomes of fixed and removable implant-assisted prostheses for the rehabilitation of atrophied distal extension mandibular ridges.

**Methods:**

Thirty partially edentulous patients with atrophied distal extension mandibular ridges were randomized to three groups (*n* = 10/group). Group ALF received long implants following alveolar ridge augmentation to support fixed restorations. Group SF received short implants to support fixed restorations. Group OVD received two long implants to support a removable partial denture. The plaque index (MPI), gingival index (MGI), Pocket depth (PD), implant stability (IS), and crestal bone loss (CBL) were assessed: immediately after the prosthesis insertion (T0), six months (T6), and twelve months (T12) later. All groups were assessed for patient satisfaction after 12 months using a visual analogue scale (VAS) survey.

**Results:**

Implant survival rates were 89.7%, 91.7%, and 85% in the ALF, SF, and OVD groups (Chi-square = 0.972, log-rank test, *p* =.673). The ALF and SF groups had significantly greater plaque and gingival scores (*P* <.05) than the OVD group At T6 and T12. The ALF group exhibited significantly higher PD and IS values (*P* ˂ 0.05) compared to the OVD group At T6 and T12. The OVD group had the greatest significant CBL values (*P* <.05) at T6 and T12, whereas the SF group presented the lowest significant values. The ALF and SF groups showed the highest significant satisfaction levels (*P* <.05) regarding appearance, esthetics, retention, stability, chewing, bolus quality, and occlusion. On the other hand, the OVD group expressed the highest significant satisfaction (*P* <.05) with surgery, healing, and cleaning.

**Conclusions:**

Fixed restorations supported by either short implants or long implants inserted in augmented bone are equally successful in the rehabilitation of the atrophic posterior mandible, with improved patient satisfaction than implant-assisted partial dentures. Nevertheless, the SF group had favorable peri-implant soft tissue health and decreased marginal bone loss compared to the ALF group. Conversely, implant-assisted partial overdentures demonstrated favorable peri-implant soft tissue health and increased patient satisfaction regarding surgery and healing compared to fixed restoration.

**Trial registration:**

Current Trial NCT05978115 (28/07/2023) “Retrospectively registered”.

**Supplementary Information:**

The online version contains supplementary material available at 10.1186/s12903-025-06286-7.

## Introduction

The prosthodontic management of partially edentulous patients with atrophied distal extension mandibular ridge presents significant challenges in clinical practice [1–3]. Removable partial dentures (RPDs) were the only traditional treatment option for a long time. Nevertheless, RPDs present several challenges, such as reduced chewing efficiency, compromised aesthetics, and problems related to retention and stability [4]. Furthermore, the lack of distal support in distal extension-RPDs causes harmful stresses to be transferred to the supporting tissues during chewing, resulting in detrimental changes in both abutment teeth and edentulous areas [3, 4]. As a result, RPDs are associated with lower patient satisfaction and continued resorption of the posterior ridge [5, 6].

Implant-supported distal extension prostheses offer potential benefits, including adequate posterior support and eliminating unesthetic retentive clasps. Furthermore, It reduces harmful horizontal stresses that could jeopardize the abutment teeth while providing support and retention. Nevertheless, several factors might complicate the clinical placement of implants, such as alveolar bone loss, inadequate bone volume, and closeness to the inferior alveolar nerve [7–9]. Bone augmentation of the alveolar ridge may be required to facilitate appropriate implant placement within adequate bone quantity and quality. This method is commonly utilized when inserting implants into atrophied posterior mandibular ridges [10]. Various surgical techniques have been used to reconstruct the alveolar ridge, such as osteodistraction, bone grafting, split-ridge osteotomy for lateral expansion, and guided bone regeneration (GBR). These methods may be used individually or in combination with grafting materials [11–13]. Autogenous bone grafts are often considered the most reliable and effective method, especially for enhancing the vertical alveolar ridge. Over the last 15 years, intraoral harvesting of autogenous bone grafts has become a standard, predictable, and safe surgical practice [14]. However, some hazards are still associated with these procedures, such as soft tissue necrosis with graft exposure and insufficient revascularization of the mandibular cortical graft, resulting in increased resorption of the grafted area [15, 16].

Short implants could be considered an alternative treatment for supporting prostheses in situations with insufficient bone above the inferior alveolar nerve [17]. Carosi et al. [18] demonstrated that the survival rates of either short implants or long implants inserted in the posterior mandible after ridge augmentation were comparable. Compared to the more invasive surgical procedures, short implants provide several benefits, such as simplicity, cost-effectiveness, shorter treatment duration, and reduced patient morbidity.

The final implant prosthesis selection should consider the patients’ preferences, economic status, compliance, maxillomandibular relationship, hygienic maintenance, and ridge anatomy. There are two types of final prostheses: (1) fixed prostheses and (2) implant-assisted overdentures. The fixed prosthesis’s overlap across the alveolar ridge may hinder insertion and complicate patient hygiene practices [19, 20]. However, utilizing fixed restorations on short implants without bone augmentation may lead to a high crown-implant ratio and challenges with hygienic maintenance, ultimately resulting in implant instability [21].

There is a lack of research comparing the clinical outcomes of different treatment methods for rehabilitating atrophied posterior mandibular ridges. This makes it challenging to determine the most effective treatment for preserving peri-implant tissue health and ensuring patient satisfaction. This clinical trial compared the clinical outcomes of three different treatment approaches addressing atrophied posterior mandibular ridges. These approaches include (1) fixed restoration supported by three long implants placed simultaneously after ridge augmentation, (2) fixed restoration supported by one long implant and two short implants, and (3) Overdentures supported by one long implant placed anterior to the mental foramen. The null hypothesis suggested that there would be no statistically significant difference in clinical outcomes among the three different treatment approaches.

## Materials and methods

### Patients’ selection and study design

Thirty partially edentulous patients with atrophied distal extension mandibular ridges were involved in a three-arm randomized controlled clinical trial. From September 2022 to January 2023, the study examiners selected the patients at the Prosthodontics Department outpatient clinic, Faculty of Dentistry, Mansoura University, Egypt. A power analysis was conducted utilizing computer software (G* Power) to determine the appropriate sample size based on the results of El Haron et al. study [22]. The authors reported a significant difference in peri-implant pocket depth after one year between three different groups using a similar study design. Using the one-way ANOVA, the sample size calculation resulted in 21 patients (7 patients in each group). The total sample size was increased to 30 (10 patients per group) by adding 9 more patients to account for potential dropouts [Pooled SD = 0.38, Effect size = 1.13, type I error (α) = 0.05, type II error (β) = 0.99]. All patients received detailed instructions prior to obtaining informed consent. The Ethics Committee of the Faculty of Dentistry at Mansour University in Egypt approved the study protocol (A01020822). Furthermore, the study was registered on ClinicalTrials.gov (NCT05978115) and complied with CONSORT criteria for clinical research.

The inclusion criteria are as follows: (1) mandibular partially edentulous patients (Kennedy class I or II), with remaining anterior teeth and the opposing arch consisting of either natural or artificial teeth, (2) 6–8 mm of residual bone height above the inferior alveolar nerve, (3) adequate inter-arch space between the occlusal plane and soft tissue (at least 8 mm) [23]. The exclusion criteria are as follows: (1) serious systemic disorders that could interfere with osteointegration or complicate surgical operations, (2) poor oral hygiene, (3) smoking habits, and (4) uncontrolled diabetic patients. The patient’s demographic information was documented, and both clinical and radiographic evaluations were performed [22].

The examiners used a computer-based Excel program to randomly allocate the patients into three groups, with ten patients in each group, using balanced randomization. This randomization technique guarantees that the groups are comparable regarding their baseline characteristics before any treatment is administered. Group ALF included ten participants who received a screw-retained fixed restoration supported by three long implants placed following vertical mandibular ridge augmentation. Group SF included ten participants who received a screw-retained fixed restoration supported by one long implant in the 1st premolar area distal to the canine and two short implants in the 2nd premolar and molar areas. Group OVD comprised ten participants who received partial overdentures assisted by long implants inserted in the 1st premolar area, distal to the canine areas.

### Surgical procedures

Initial treatment includes oral hygiene instruction, scaling, root planning, and extracting hopeless teeth to reduce periodontal pathogens over time. Medical history and clinical examination were completed for all participants. The mandibular alveolar bone’s height, width, and density were assessed using CBCT (i-CAT). All groups were provided with interim acrylic RPDs with wrought wire clasps. In the ALF group, the mandibular ramus was used as the source of an autogenous bone block for the grafting procedures utilizing a Piezo surgery device [23]. The harvested bone was cautiously refined using a bone scraper (Safe-scraper Twist curved, Meta) to produce 1-mm-thick blocks and particulate bone. The alveolar crestal bone was decorticated at several locations using a small round bur to expose the underlying native bone. After that, the bone block was secured to the alveolar ridge using micro screws to facilitate ridge augmentation. This grafting technique utilized autogenous bone in conjunction with autogenous bone chips. The postoperative prescription consists of antibiotics (625 mg amoxicillin + 125 mg clavulanic acid) and mouthwash with chlorhexidine digluconate 0.2%, given twice a day for seven days. Moreover, non-steroidal anti-inflammatory medications (chymotrypsin 25 mg) and analgesics (ketoprofen 10 mg) were administered one day prior to surgery and maintained for five subsequent days. To reduce postoperative edema after surgery, the patients were instructed to apply ice packs. One peri-oral surgeon (Elkashty A.) performed surgical procedures. Following a 4–5-month period for osseous healing, mucoperiosteal flaps were elevated for the placement of dental implants. Long implants measure 10–13 mm in length, whereas short implants measure 5.5–8 mm in length. J Dental Care Implant, JD Evolution Plus, Italy, were utilized for all groups.

**Fig. 1:**
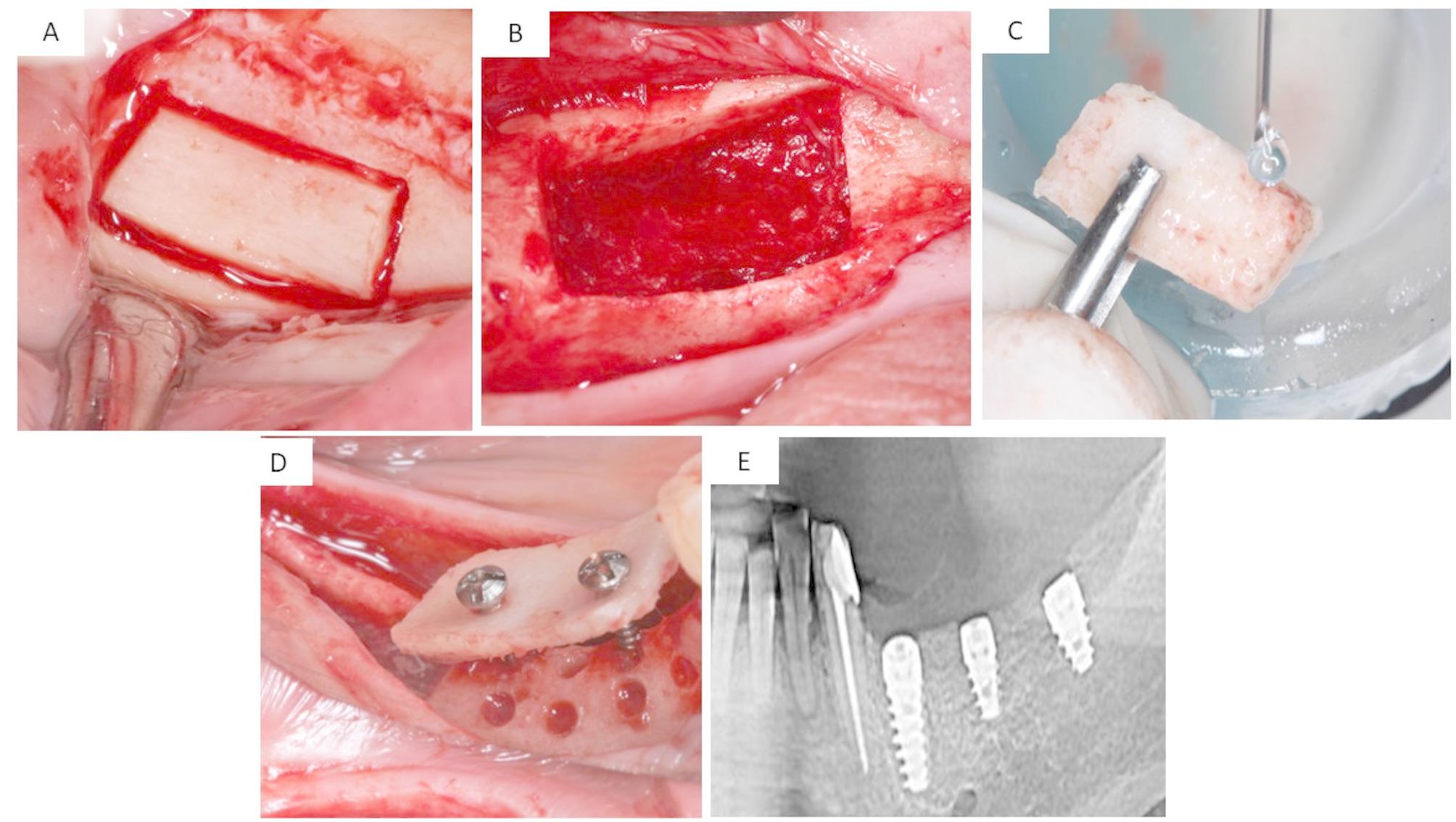
The ALF group: (**A**) Bone block preparation in one patient, (**B**) Mandibular site after bone block preparation was completed, (**C**) The resulting bone block, (**D**) Fixation of the bone block harvested from the ramus with micro screw, (**E**) Postoperative radiograph of the long implants placed after ridge augmentation,

**Fig. 2:**
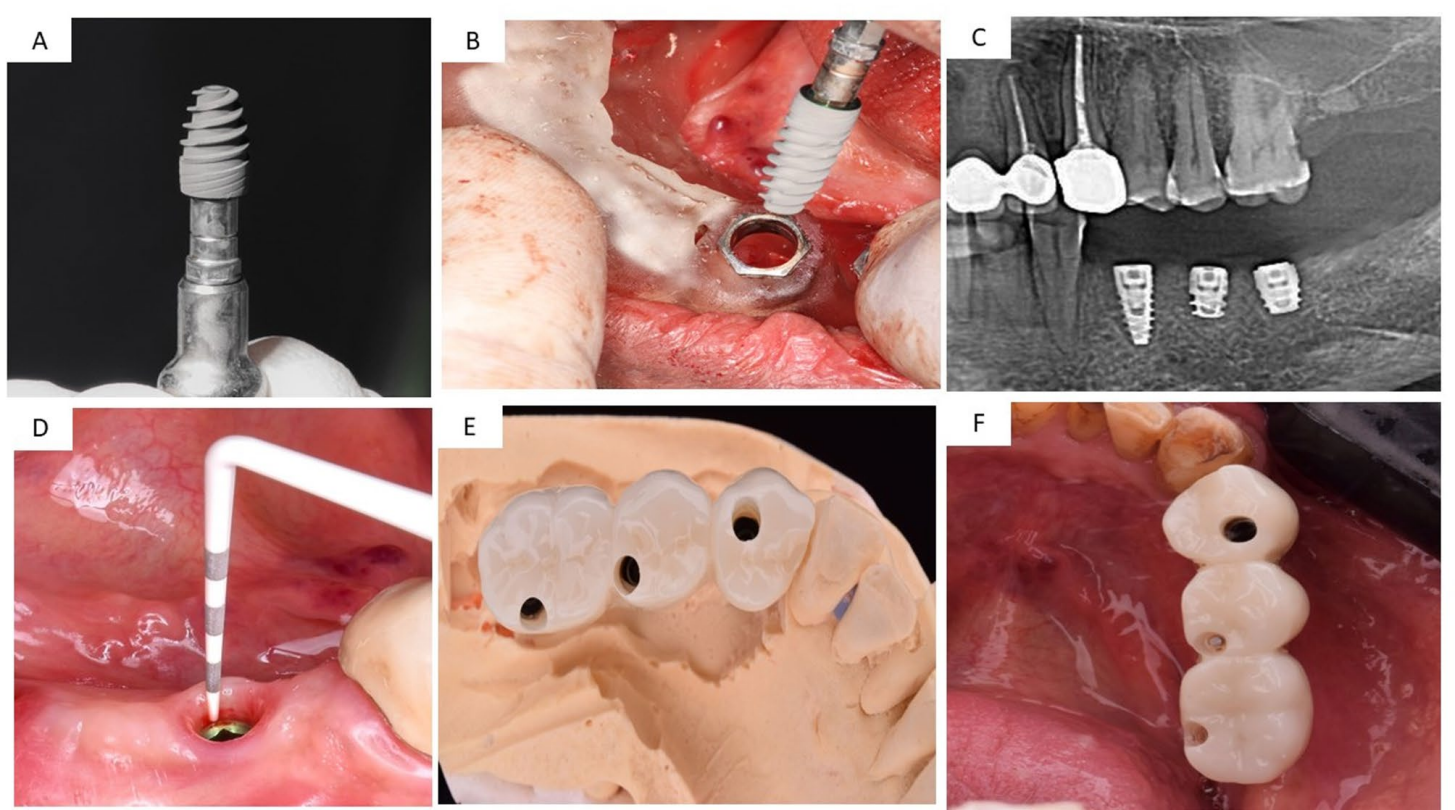
The SF group: (**A**) The short implant fixture mounted to the handpiece, (**B**) Insertion of the short implant using surgical template, (**C**) Postoperative radiograph of short implants, (**D**) Calibrated plastic periodontal probe assessing peri-implant tissues, (**E**) Occlusal view of the fixed screw-retained restoration on the cast, (**F**) Occlusal view of the fixed screw-retained restoration in the patient mouth

**Fig. 3:**
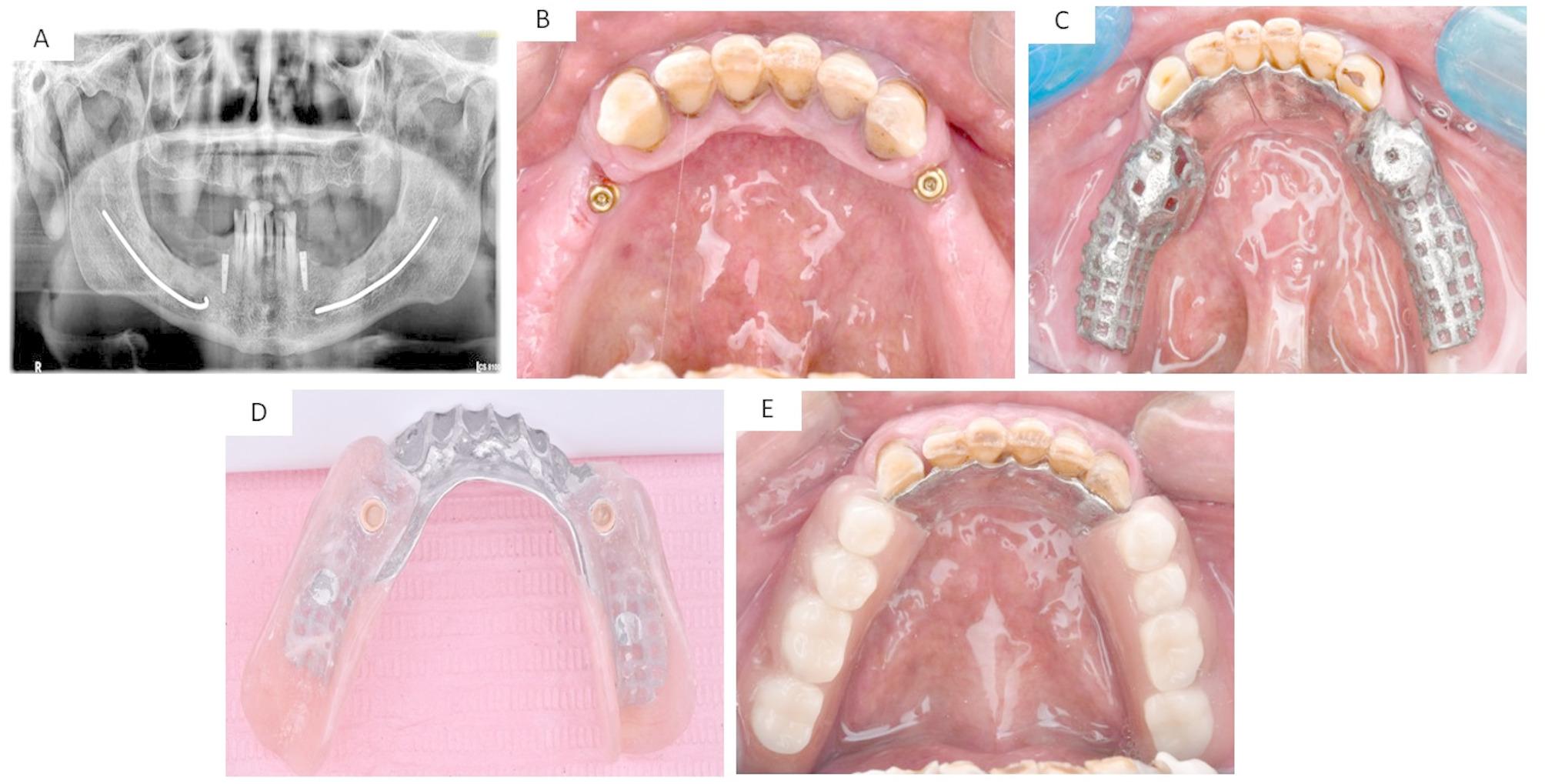
The OVD group: (**A**) Postoperative radiograph of implants, (**B**) Locator attachment in patient mouth, (**C**) The cast metal framework in patient mouth, (**D**) Fitting surface of the overdenture with nylon inserts inside the metal housings, (**E**) Occlusal view of implant assisted overdenture in the patient mouth

The treatment plan for each group was virtually designed to guarantee the optimal position, length, width, and distribution of implants. One prosthodontist (Elsyad, M) verified the treatment plan for all groups. The treatment plan was utilized to produce a stereolithographic surgical template with three sleeves that were aligned with the designated implant locations. The surgical procedure was performed in two stages using a conventional loading protocol. Group ALF received three long implants: one in the first premolar region and two in the second premolar and first molar regions (bilateral distal extension cases, *n* = 5; unilateral distal extension cases, *n* = 5) [Figure 1]. The SF group received one long implant in the first premolar area and two short implants in the second premolar and first molar regions (bilateral distal extension cases, *n* = 3; unilateral distal extension cases, *n* = 7) [Figure 2]. The OVD group received two long implants inserted in the first premolar regions and distal to the canine area (bilateral distal extension cases, *n* = 10) [Figure 3]. The patients were advised to apply cold packs to minimize swelling after surgery, follow a soft diet for six weeks, and maintain good oral hygiene. Following surgery, patients were given an intramuscular injection of Voltaren (75 ml IM/IV, NOVARTIS, Egypt). Subsequently, they were prescribed antibiotics (Augmentin 1gm for 5–7 days) along with Ketoprofen 50 mg (three times daily for three days) to relieve pain and swelling.

### Prosthetic procedures

The implants were uncovered after (three months for SF and OVD groups, and 6 months for ALF group) and healing abutments were inserted for two weeks. Metal–ceramic screw-retained FPDs were utilized for ALF and SF groups. An open tray impression was obtained utilizing an additional silicone impression material (Silaxil, LASCOD Florence). Duralay resin (Duralay, Reliance Dental) was applied to connect the long impression posts and to prevent transfer coping displacement during impression removal.

The impression was poured using dental stone following the attachment of the analogs to the impression posts. CAD/CAM software (Ceramill Map 400, Amann Girrbach) designed screw-retained fixed restorations from scanned casts. Castable resin (GC Pattern Resin, GC) was used to print the restoration designs. The printed resin was then cast in a cobalt-chromium alloy and assessed for passive fit in the patient’s mouth. The cobalt chromium was coated with an opaquer layer, and the porcelain was fired and glazed (VITA Zahnfabrik). Group function occlusal scheme was used for both ALF and SF groups. For the OVD group, the cast was surveyed, and the mouth preparations were completed, including proximal plates, occlusal rests, and cingulum rests. Subsequently, the casts were scanned, and CAD/CAM software was utilized to produce removable restorations in castable resin. Permanent metallic removable partial dentures (RPDs) were made using a lingual plate major connector. Locator abutments (Zest Anchors Inc.) were mounted to implants. The metal housings and blocking rings were fixed to the abutments, and the RPD denture bases were properly relieved to accommodate them. The metal housings were picked up onto the fitting surface of RPDs using auto-polymerized acrylic resin in centric occlusion. Pink nylon inserts with medium retention were utilized. The canine-guided occlusal concept was applied in all groups [24], and patients received instructions on maintaining proper oral hygiene and consistent home care after getting the prostheses.

### Study measurement

The clinical evaluation was conducted at three different time points: immediately after the insertion of the prosthesis (T0), six months later (T6), and twelve months later (T12). A calibrated prosthodontist (Raffat E) measured the clinical parameters. The assessment included the use of the Modified Plaque Index (MPI) [25], Modified Gingival Index (MGI) [25], and Pocket Depth (PD) [26] to assess peri-implant tissue health. In addition, evaluations were made for Crestal Bone Loss (CBL) [27], implant stability [26], implant survival rate [28], and patient satisfaction [29, 30]. The peri-implant tissue was assessed using a calibrated plastic periodontal probe with 0.5 N probing force. The pocket depth (PD) was measured in mm from the gingival margin to the deepest point of the probing.

Each implant’s buccal and lingual surfaces were measured for MPI, MGI, and PD in the ALF and SF groups, while the mesial, distal, buccal, and lingual surfaces were measured for the OVD group. Marginal bone loss was assessed on the mesial and distal surfaces of the implant using standardized long cone periapical radiography obtained through a digital device (Digora, Soredex). The position of the plastic film holder was standardized by inserting a clear acrylic jig between the artificial teeth in centric occlusion. The height of the bone was measured by calculating the distance from the implant shoulder to the first bone-to-implant contact (DIB values). Crestal bone loss (CBL) was determined by subtracting the DIB at T6 and T12 from the DIB at T0. To compensate for magnification errors, the actual values of CBL were obtained using the actual dimensions of the implants. Implant stability was assessed using the Osstell device (Osstell, Integration Diagnostics), with the implant stability quotient (ISQ) serving as the measurement unit. The implant was considered successful if it met the success criteria (no pain, absence of implant mobility, and less than 1.5 mm marginal bone loss). Implants that did not meet success criteria but were still functioning and did not require removal were considered survived. The survival rate of the implants was assessed through Kaplan Meier analysis in this study. All groups were assessed for patient satisfaction after 12 months utilizing a visual analogue scale (VAS) survey. The survey covered appearance, comfort, ease of chewing and cleaning, ease of speech, overall prosthesis satisfaction compared to natural teeth, food bolus quality, occlusion retention and stability, and surgery and healing satisfaction. The participants’ satisfaction was assessed on a 100 scale, with 0 indicating no satisfaction and 100 representing complete satisfaction. The survey was translated to Arabic through a process of forward and backward translation, and its validity was confirmed in a previous study [30].

### Statistical analysis

The SPSS^®^ software version 22 (SPSS Inc., Chicago, IL, USA) was used to conduct the analysis. A biostatistician with expertise in dentistry analyzed the data without knowledge of the group codes. The baseline characteristics for all groups were compared using one-way ANOVA for numerical data and the Chi-square test for categorical (binary) variables. Median (minimum-maximum) values were used to report non-parametric data (PI, GI, VAS), and mean ± SD values were used for parametric data (PD, ISQ, CBL). The Kruskal-Wallis and Mann-Whitney tests were used to compare medians of MPI, MGI, and VAS between groups. The Friedman test was utilized to determine significant differences in medians between observation times for non-parametric data, subsequently applying the Wilcoxon signed ranks test for comparisons within the same group. Means of parametric data were compared between groups and observation times using a two-way mixed ANOVA, with pairwise comparisons conducted using the Bonferroni test. Survival rates were evaluated using Kaplan-Meier analysis. A comparison of survival rates between groups was made using the log-rank test. All statistical tests were set at a significant level of 5%.

## Results

**Fig. 4:**
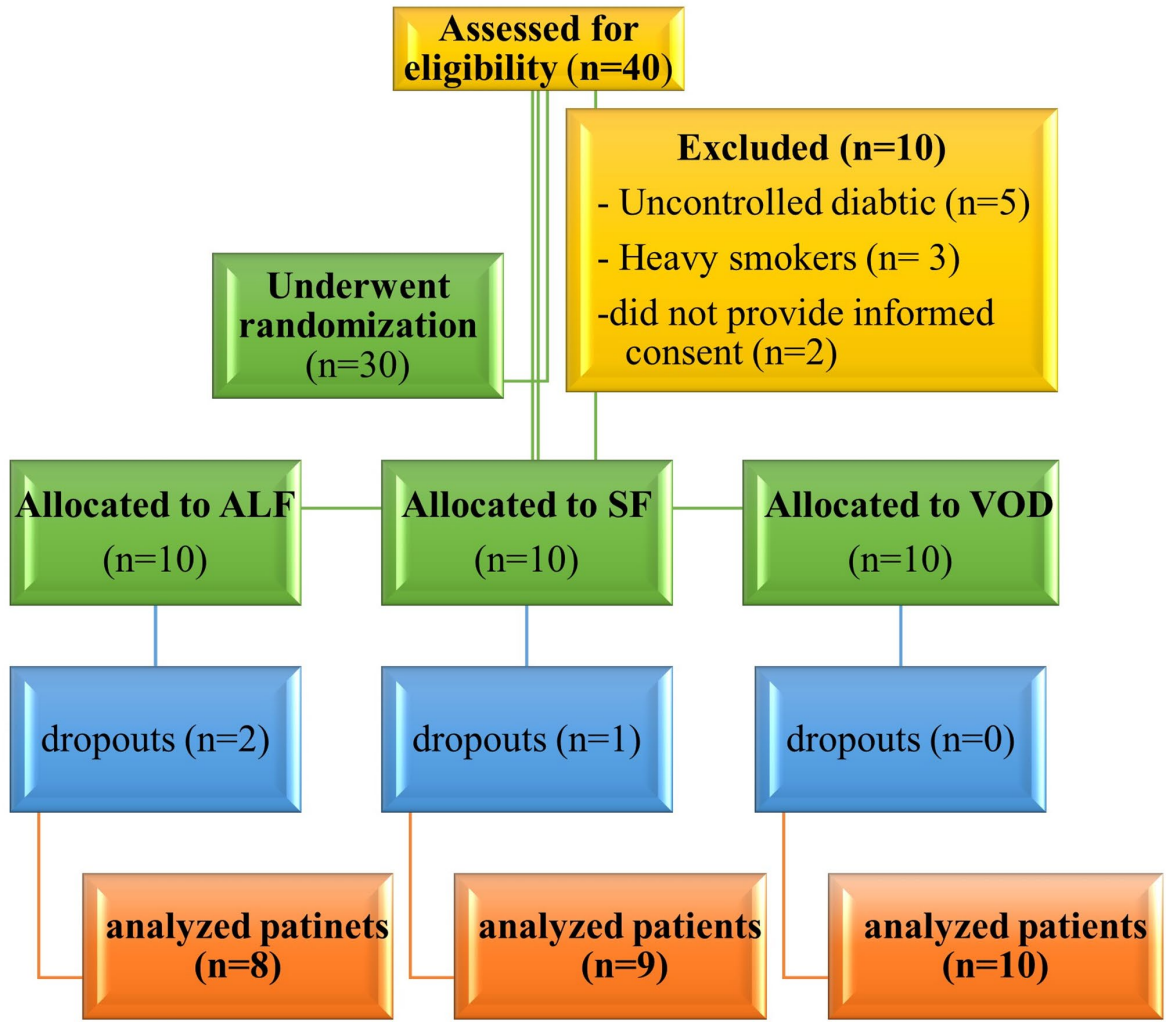
Patient Flow Diagram

The baseline characteristics for all groups are presented in Table 1. The study eliminated three participants, two from the ALF group and one from the SF group (Fig. 4). In the ALF group, one participant refused to participate in the follow-up, while another experienced a serious illness. In the SF group, one participant could not be reached for an examination.

**Table 1:** Baseline characteristics for all groups

	ALF	SF	OVD	*P* value
Mean Age (years) X ± SD	57.62 ± 2.51	62.66 ± 2.48	60.00 ± 4.35	0.157
Gender (male/female) Frequency	4/6	5/5	6/4	0.368
Mean remaining bone height above mandibular canal in (mm) X ± SD	6.85 ± 1.3	7.33 ± 0.98	7.5 ± 0.45	0.472
Periodontal disease Frequency	4	3	5	0.847

### Implant Survival

Within the ALF group, four implants (in two participants) failed: two during implant uncovering, one after six months, and one after a year, leading to an 89.7% implant survival rate. Three implants (in two participants) in the SF group failed six months after implant loading, leading to a 91.6% implant survival rate. In the OVD group, two participants experienced three implant failures, with one occurring at the uncovering stage and two after six months, leading to an 85% implant survival rate. The survival rate was calculated after excluding the dropout patients. The Kaplan-Meier analysis showed no significant difference in implant survival across all groups (Chi-square = 0.972, log-rank test, *p* =.673).

### Peri-implant health

MPI and MGI increased significantly across all groups over time. The ALF and SF groups had significantly greater plaque and gingival scores than the OVD group at T6 and T12 (Table 2). There was a significant increase in PD, IS, and CBL across all groups over time. Comparatively, the ALF and SF groups exhibited significantly higher PD and IS than the OVD group. At T6 and T12, the OVD group demonstrated the highest CBL, while the SF group showed the lowest values. Moreover, the ALF group revealed significantly higher CBL compared to the SF group (Table 3).

**Table 2:** Comparison of plaque (MPI) and gingival (MGI) scores between observation times and groups

Measurements	Groups	Observation Periods (Months)				Friedman test *P* value	
		T0 M (Min-Max)	T6 M (Min-Max)	T12 M (Min-Ma)x			
Modified Plaque Index (MPI)	ALF	2.00 ^A, a^ (1.00–3.00)	2.00 ^A, a^ (1.00–3.00)	3.00 ^A, b^ (2.00–3.00)		< 0.001*	
	SF	1.00 ^B, a^ (1.00–2.00)	2.00 ^A, b^ (1.00–2.00)	2.00 ^A, c^ (2.00–3.00)		< 0.001*	
	OVD	1.00 ^B, a^ (1.00–3.00)	1.00 ^B, b^ (1.00–2.00)	1.00 ^B, b^ (1.00–2.00)		< 0.001*	
	Kruskal-Wallis test, *P* value	0.085	< 0.001*	< 0.001*			
Modified Gingival Index (MGI)	ALF	2.00 ^A, a^ (1.00–3.00)	2.00 ^A, b^ (1.00–3.00)	2.5.00 ^A, c^ (2.00–3.00)		< 0.001*	
	SF	1.00 ^B, a^ (1.00–2.00)	2.00 ^A, b^ (1.00–3.00)	2.00 ^A, c^ (2.00–3.00)		< 0.001*	
	OVD	2.00 ^A, a^ (1.00–2.00)	1.00 ^B, b^ (1.00–3.00)	1.00 ^B, b^ (1.00–3.00)		0.002*	
	Kruskal-Wallis test, *P* value	0.005*	˂0.001*	< 0.001*			

M; median, min; minimum, max; maximum, * p is significant at 5% level. Different upper-case letters in the same column indicate

a significant difference between each 2-groups (Mann Whitney test, *p* <.05). Different lower-case letters in the same raw indicate a significant difference between each 2-observation time (Wilcoxon signed ranks test, *p* <.05)

**Table 3:** Comparison of probing depth (PD), implant stability (IS), and crestal bone loss (CBL) between observation times and groups

**Measurements**	**Groups**	**Observation Periods (Months)**				Mixed ANOVA test *P* value
		T0 X ± SD	T6 X ± SD	T12 X ± SD		
Probing Depth (PD)	ALF	2.03 ±.117 ^A, a^	2.36 ±.069 ^A, b^	2.57 ±.061 ^A, c^		<.001*
	SF	1.88 ±.042 ^B, a^	2.04 ±.048 ^B, b^	2.13 ±.063 ^B, c^		<.001*
	OVD	1.76 ±.057 ^C, a^	1.58 ±.056 ^C, b^	1.42 ±.048 ^C, c^		<.001*
	Mixed ANOVA test, *P* value	<.001*	<.001*	<.001*		
Implant Stability (IS)	ALF	74.65 ± 1.90 ^A, a^	74.91 ± 2.54 ^A, a^	75.07 ± 1.26 ^A, a^		<.001*
	SF	73.46 ± 1.35 ^A, a^	71.31 ± 2.15 ^B, b^	69.18 ± 2.21^B, c^		<.001*
	OVD	62.02 ± 2.63 ^B, a^	57.15 ± 1.87 C^, b^	53.51 ± 2.35 ^C, b^		<.001*
	Mixed ANOVA test *P* value	˂.05*	˂.001*	<.001*		
Crestal Bone Loss (CBL)	ALF	-	.57 ±.021 ^A, a^	.78 ±.020 ^A, b^		<.001*
	SF	-	.32 ±.024 ^B, a^	.52 ±.029 ^B, b^		<.005*
	OVD	-	.78 ±.026 ^C, a^	.96 ±.033 ^C, a^		<.001*
	Mixed ANOVA test *P* value		<.001*	<.001*		

X; mean, SD; standard deviation, * p is significant at 5% level. Different upper-case letters in the same column indicate

a significant difference between each 2-groups (Bonferroni test, *p* <.05). Different lower-case letters in the same raw indicate a significant difference between each 2-observation time (Bonferroni test, *p* <.05)

### Patients’ satisfaction

The ALF and SF groups showed the highest satisfaction levels regarding appearance, esthetics, retention, stability, chewing, bolus quality, and occlusion. On the other hand, the OVD group expressed the highest satisfaction with surgery, healing, and cleaning (Table 4).

**Table 4:** Results of visual analogue scale for all groups

	ALF X ± SD, M (min-max)	SF X, M (min-max)	OVD X, M (min-max)	Kruskal Wallis test
Appearance	85 ± 5.0^a^ 85(80–90)	80 ± 4.9 ^a^ 80(75–85)	40 ± 5.0 ^b^ 40(40–50)	0.048*
Comfort with prosthesis	86.25 ± 4.7 ^a^ 87.5(80–90)	81.66 ± 18.9 ^a^ 70(40–75)	40 ± 7.07 ^b^ 40(35–45)	0.039*
Ease of chewing	88.33 ± 2.8 ^a^ 90(85–90)	75 ± 5.0 ^a^ 75(80–90)	38.33 ± 3.0 ^b^ 40(35–40)	0.026*
Ease of cleaning of prosthesis	65 ± 5.0 ^a^ 65(60–70)	65 ± 4.9 ^a^ 65(60–70)	76.66 ± 2.8^b^ 75(75–80)	0.048*
Ease of speaking	85 ± 5.0 ^a^ 85(80–90)	78.33 ± 5.7 ^a^ 75(75–80)	35 ± 5.0 ^b^ 35(30–40)	0.043*
Embarrassing	81.66 ± 2.8 ^a^ 80(80–85)	76.66 ± 3.0 ^a^ 75(75–80)	35 ± 5.0 ^b^ 35(30–40)	0.034*
General satisfaction with mandibular prosthesis compared to teeth	76.66 ± 5.7 ^a^ 80(70–80)	81 ± 2.9 ^a^ 80(80–85)	28.33 ± 7.6 ^b^ 30(20–35)	0.039*
Occlusion of prosthesis	85 ± 5.0 ^a^ 85(80–90)	75.33 ± 5.7 ^a^ 75(70–80)	37.5 ± 5.0 ^b^ 40(30–40)	0.031*
Prosthesis apart of you	85 ± 5.0 ^a^ 85(80–90)	81.66 ± 3.0 ^a^ 80(80–90)	33.33 ± 2.8 ^b^ 35(30–35)	0.026*
Quality of bolus	83.33 ± 3.8 ^a^ 85(80–85)	73.33 ± 2.8 ^a^ 75(70–75)	35 ± 5.0 ^b^ 35(30–40)	0.026*
Retention and stability of prosthesis	85 ± 5.0 ^a^ 85(80–90)	80.8 ± 5.5 ^a^ 80(75–85)	35 ± 5.0 ^b^ 35(30–40)	0.048*
Satisfaction with surgery and healing	38.33 ± 2.8 ^a^ 40(35–40)	70 ± 5.0 ^b^ 70(65–75)	65 ± 4.5 ^b^ 65(60–70)	0.047*

X: mean, SD; standard deviation; M: median, min: minimum, max: maximum. *P value is significant at 5% level. Different letters in the same raw indicates a significant difference between each 2-groups (Mann Whitney test, *p* <.05)

## Discussion

The ALF and SF groups had a significantly higher survival rate with fixed restoration compared to the OVD group. The higher number of implants in these groups may enhance effective load distribution, thereby improving their survival rates [31]. The lack of difference in implant survival rates between the ALF and SF groups is consistent with previous studies [32, 33], that observed no significant difference in outcomes between short and long implants inserted in atrophic posterior mandibles after ridge augmentation. In contrast, other studies reported that standard-length implants inserted into augmented bone demonstrate higher survival rates compared to short implants over a ten-year follow-up period [34, 35]. The lower implant survival rates in the OVD group are consistent with the conclusions of El Haroon et al. [22]. and Ortiz-Puigpelat et al. [36]. This can be explained by the increased stresses transferred to the implants resulting from their mesial placement, which leads to a longer cantilevered distal extension. Furthermore, the locator attachments exhibit a limited range of motion, potentially leading to increased stress on the implants as the denture rotates during use [37].

The ALF group demonstrated significantly higher MPI, MGI, and PD scores compared to other groups. This could be explained by the postoperative inflammation, discomfort, and the reduced oral hygiene during the healing phase. Furthermore, the inaccessibility of posterior implants, irretrievability of fixed restorations, and cleaning concerns may contribute to plaque buildup and gingival inflammation in the ALF and SF groups. The lowest MPI, MGI, and PD scores in the OVD group are consistent with the findings of Al Haroon et al. [22]. They reported that implant-assisted RPDs demonstrated more favorable peri-implant soft tissue health than implant-assisted FPD. The overdenture prosthesis is designed to be easily removable, allowing patients to maintain better oral hygiene and cleanliness [38]. Additionally, the implants in the OVD group are easier to clean owing to their anterior positioning than the posterior implants in the ALF and SF groups [37]. Additionally, the self-cleaning capability of the locator attachments can enhance peri-implant tissue health by decreasing plaque buildup and gingival irritation. As a result of increased gingival inflammation and plaque accumulation, the ALF group had deeper peri-implant pockets than the OVD group [22].

The ALF and SF groups had the highest implant stability (ISQ = 75.07 ± 1.26, and 69.18 ± 2.21 respectively), while the OVD group had the lowest stability (ISQ = 53.51 ± 2.35).The increased stability in the ALF group may be attributed to the greater surface area of the long implant and a reduced crown root ratio, leading to improved bone contact compared to the smaller surface area and high crown root ratio of short implants [39–41]. Similarly, Chun Wu et al. [42] reported that long implants exhibit enhanced primary stability compared to short implants by promoting intraosseous anchorage. The lower implant stability observed in the OVD group may be due to the increased stresses transmitted to the implants [36]. Implant length significantly influences load transfer at the bone-implant interface. An increase in implant length enhances initial stability due to the overall increase in total surface area. Furthermore, it offers resistance to torque and shear forces while achieving stabilization [41–44].

The ALF group showed considerable alveolar bone loss compared to the SF group, attributed to the following reasons; first, the bone blocks inserted into the mandible may undergo a slow revascularization process, leading to substantial marginal bone remodeling around the implant in the ALF group [23, 45]. Similarly, Pier et al. [23] and Esposito et al. [47] observed a significant increase in peri-implant marginal bone loss one year after mandibular ridge augmentation when compared to the initial baseline measurements. Secondly, the reduction in marginal bone loss in the SF group may be due to the use of splinted restorations, which could alleviate the adverse effects of high C/I ratios on peri-implant bone and distribute the occlusal stresses more uniformly across implants [46]. The increased marginal bone loss observed in the OVD group can be attributed to various biomechanical factors: the extended cantilevered distal extension prosthesis [48], the limited hinge motion of the locator during usage [37], and the mesial placement of implants [49]. All these factors could increase peri-implant stresses and contribute to increased bone loss. Similarly, Patil et al. [50] reported an increase in crestal bone loss in mandibular-assisted overdentures after one year, compared to the initial measurements. This study reported that implants exhibiting a significant increase in crestal bone loss also demonstrated greater probing depth.

The ultimate success of the dental treatment plan is significantly influenced by patient feedback. Prosthodontists must evaluate the influence of prosthesis type on patient satisfaction to make appropriate decisions in selecting the best alternative in prosthodontic rehabilitation [51, 52]. The ALF and SF groups reported higher satisfaction with appearance compared to the OVD group. Several studies similarly reported that the patients expressed greater satisfaction with fixed prostheses in terms of aesthetics compared to removable prostheses [52–54]. The decreased patient satisfaction with aesthetics in the OVD group could be attributed to increased visibility of the metal framework and the bulk of acrylic resin, resulting in lip bulging. The thickness of major connectors may interfere with the tongue and reduce patient satisfaction with speech [22]. The ALF and SF groups reported greater patient satisfaction with retention and stability because the fixed prostheses are attached to the implants with screws, leading to improved patient comfort and the perception that the prosthesis is an integral component of the patient [55]. The ALF and SF groups reported greater satisfaction level regarding chewing, bolus quality, and occlusion compared to the OVD group. This may be due to the enhanced stability, improved chewing efficiency, and better quality of bolus provided by fixed restorations [56, 57]. The results of this study are corroborated by a recent meta-analysis indicating that implant-supported fixed restorations enhance patient satisfaction regarding oral health-related quality of life more than implant-supported overdentures [58].

Patient satisfaction regarding surgery and healing was higher in the OVD group, while the ALF group reported the lowest satisfaction level. This could be attributed to the higher surgical complications in the ALF group. This explanation was consistent with several studies indicating that, mandibular ridge augmentation is technically more complex and associated with higher morbidity compared to the insertion of short implants [23, 59, 60]. The OVD group reported increased satisfaction with cleaning due to the patient’s ability to remove the prosthesis and clean the peri-implant areas. Previous study reported that overdentures are easier to clean and handle than fixed restorations supported by four implants [61].

This study could assist clinicians in determining the optimal treatment for similar clinical situations. The current study has some limitations, including a short follow-up period of only 12 months, small sample size and the administration of a patient satisfaction survey only once, after 12 months. Survey questionnaires also depend on subjective responses that can change daily, sometimes even within the same day. Additional longer follow-up periods with larger sample size are required to achieve long-term outcomes. Moreover, the decreased number of implants and their mesial positioning in the OVD group may potentially leading to biomechanical complications. Among limitations of this study is the fact that several confounding factors affecting bone loss such as bone quality and patient compliance with oral hygiene, and biting force were not investigated. In the literature, the high bite force capacity, especially in the posterior region of the mouth could increase marginal bone loss and may result in implant failure [62, 63]. A further limitation is that these findings primarily reflect the population within the geographic context of the study. The applicability of these findings to other populations with differing dietary habits or socioeconomic factors is a limitation in this study. Also, that surgeon was experienced with techniques. However, all the procedures were tested in a real clinical condition. So, the results of the trial can be generalized to wider population with similar characteristics. This study was retrospectively registered at ClinicalTrials.gov due to miscommunication issues.

## Conclusions

Within the limitations of this study, after a one-year follow-up, fixed restorations supported by either short implants or long implants placed in augmented bone are equally successful in the rehabilitation of the atrophic posterior mandible, with improved patient satisfaction than implant-assisted partial dentures. Nevertheless, the SF group had favorable peri-implant soft tissue health and reduced marginal bone loss compared to the ALF group. Conversely, implant-assisted partial overdentures demonstrated favorable peri-implant soft tissue health and increased patient satisfaction regarding surgery and healing compared to fixed restoration.

## Electronic supplementary material

Below is the link to the electronic supplementary material.

Supplementary Material 1

## Data Availability

The datasets used and/or analyzed during the current study are available from the corresponding author upon reasonable request.
