# Correction: Comparative analysis of implant survival, peri-implant health, and patient satisfaction among three treatment modalities in atrophic posterior mandibles: a randomized clinical study

**DOI:** 10.1186/s12903-025-07648-x

**Published:** 2026-01-22

**Authors:** Eman Mohamed Raffat, Mohamed Shady, Ayman Abdel Rahim Elkashty, Moustafa Abdou Elsyad

**Affiliations:** 1 Prosthodontic Department, Faculty of Dentistry, Mansoura University, Mansoura, Egypt; 2 Prosthodontic Department, Faculty of Dentistry, Benha National University, Obour, Egypt; 3 Periodontology, Oral Diagnosis and Radiology Department, Faculty of Dentistry, Mansoura University, Mansoura, Egypt

**Correction: BMC Oral Health 25, 939 (2025)**

**https://doi.org/10.1186/s12903-025-06286-7**

In this article [1], the author’s name Moustafa Abdou Elsyad was incorrectly written as Moustafa El Syad.

Incorrect author name:

Moustafa El Syad.

Correct author name:

Moustafa Abdou Elsyad.
